# Epigenetic markers for early detection of nasopharyngeal carcinoma in a high risk population

**DOI:** 10.1186/1476-4598-10-48

**Published:** 2011-05-02

**Authors:** Susanna H Hutajulu, Sagung R Indrasari, Luh PL Indrawati, Ahmad Harijadi, Sylvia Duin, Sofia M Haryana, Renske DM Steenbergen, Astrid E Greijer, Jaap M Middeldorp

**Affiliations:** 1 Faculty of Medicine/Dr. Sardjito Hospital, Universitas Gadjah Mada, Yogyakarta, Indonesia; 2 Department of Pathology, VU University medical center, Amsterdam, The Netherlands

## Abstract

**Background:**

Undifferentiated nasopharyngeal carcinoma (NPC) is strongly related to Epstein-Barr virus (EBV) infection, allowing aberrant antibodies against EBV and viral DNA load as screening tools in high risk populations. Methylation analysis in the promoter of tumor suppressor genes (TSGs) may serve as a complementary marker for identifying early cases. This study determined methylation status of multiple TSGs and evaluated whether it may improve early detection.

**Methods:**

Nasopharyngeal brushings were taken from 53 NPC patients, 22 high risk subjects and 25 healthy EBV carriers. Corresponding NPC paraffin tissue was included. DNA was bisulfite-modified preceding analysis by methylation-specific PCR (MSP). Ten TSGs were studied.

**Results:**

NPC paraffin and brushing DNA revealed an 81.8% concordance so that MSP analysis was done using either one of both specimens. NPC samples showed methylation for individual TSGs (DAPK1 79.2%, CDH13 77.4%, DLC1 76.9%, RASSF1A 75.5%, CADM1 69.8%, p16 66.0%, WIF1 61.2%, CHFR 58.5%, RIZ1 56.6% and RASSF2A 29.2%). High risk individuals, having elevated EBV IgA and viral load, showed high frequency of methylation of CDH13, DAPK1, DLC1 and CADM1, but low frequency of methylation of p16 and WIF1 and undetectable methylation of RASSF1A, CHFR, RIZ1 and RASSF2A. Healthy subjects showed similar patterns as high risk individuals. A combination of RASSF1A and p16 gave good discrimination between NPC and non-NPC, but best results were combined analysis of five methylation markers (RASSF1A, p16, WIF1, CHFR and RIZ1) with detection rate of 98%.

**Conclusion:**

Multiple marker MSP is proposed as a complementary test for NPC risk assessment in combination with EBV-based markers.

## Background

Nasopharyngeal carcinoma (NPC) is highly prevalent in Southern China and South-East Asia. The incidence of NPC in Indonesia is 6.2 cases/100,000 population per year representing the fourth most common cancer [1]. Based on size of the Indonesian population it is estimated that 13,000 new cases of NPC occur annualy. Early detection is needed to improve patient survival since the majority of cases are currently diagnosed at late stage.

Given the close link between NPC and Epstein-Barr virus (EBV) infection, detection of characteristic antibodies against EBV and elevated viral load has been proposed as useful screening tool [2-4]. Recently, we started a screening protocol in the Yogyakarta region recruiting high risk patients with chronic problems in the head and neck area and testing them using EBV-based assays. Although this study is still ongoing, current observations indicate that neither method provides an adequate stand-alone marker for detecting NPC as a primary screening test due to their relative low positive predictive value (PPV) (Hutajulu et al., unpublished data) confirming other reports [5,6]. The low PPV might be due to the large number of subjects presenting with elevated EBV antibody levels and viral load that showed no clinical mass. Indeed, antibody levels against EBV were shown to be elevated for as long as ten years before tumor presentation [4]. As a consequence, recruitment of subjects by EBV-based markers alone would require long term monitoring. CT-scan examination and nasopharyngeal biopsy are needed for clinical confirmation of tumor presence. This would imply that a huge number of people would need detailed examination resulting in unacceptably high costs. It is therefore essential to define additional NPC progression markers for better selection of high risk patients.

Exploration of altered cellular genes involved in NPC development may provide a complementary test for risk assessment in EBV infected individuals. Promoter DNA methylation is widely considered to be an important epigenetic mechanism in carcinogenesis. In NPC, gene silencing by promoter methylation has been shown for multiple tumor suppressor genes (TSGs). Each TSG potentially contributes to the multistep oncogenesis including alterations of apoptosis, cell cycle and mitotic checkpoint regulation, intracellular adhesion, cytoskeleton organization, and Wnt-signalling pathway [7-9]. Therefore, in the present study we determined the frequency of promoter DNA methylation of multiple genes in Indonesian NPC cases. These TSGs were proven to be frequently methylated in NPC from other ethnic groups [10-16]. To analyse their potential value for detection of early-stage NPC, we compared the methylation pattern of NPC patients, healthy EBV carriers and a high risk population. The latter group consisted of patients with chronic head and neck complaints showing elevated EBV markers. Furthermore, we analysed an additional TSG, the myelin and lymphocyte-associated protein (MAL), which has been proven methylated in head and neck cancer [17] but has not been tested in NPC. More importantly, MAL gene is frequently methylated in cervical cancer indicating its role in virus-related epithelial malignancy [18].

## Results

### EBV IgA ELISA seroreactivity

**Figure 1:**
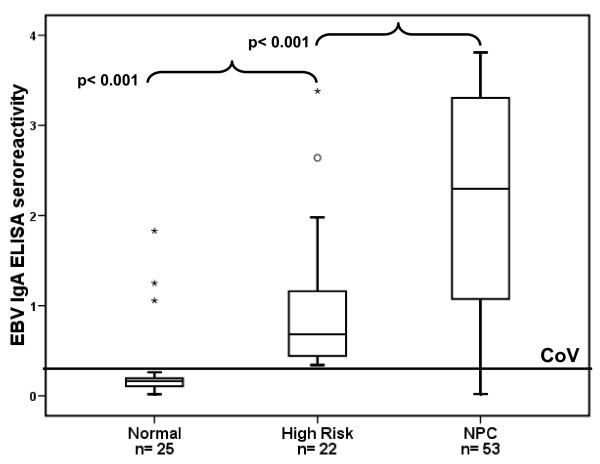
**Box plot of seroreactivity of IgA/[EBNA-1+VCA-p18] ELISA by group (normal, high risk and NPC) with cut-off value of 0.354**. The seroreactivity in NPC and high risk group was significantly higher than that in normal subjects (Mann-Whitney U test all p < 0.001). NPC = nasopharyngeal carcinoma, CoV = cut-off value.

The antibody reactivity against EBV antigens was determined for all NPC, high risk and normal subjects. Elevated responses were seen in 49 NPC cases (92.5%) and 3 normal subjects (12%). A high risk group was defined (n = 22) by selecting patients who visited the ear, nose and throat (ENT) department and showed chronic symptoms in the head and neck region (n = 212). All selected subjects from this population had elevated EBV IgA ELISA seroreactivity and detectable viral load in their nasopharyngeal brushings. The ELISA results of the three groups are displayed in Figure 1. The seroreactivity of NPC patients and high risk individuals was higher than that of healthy subjects and NPC cases showed higher responses compared to the high risk individuals (Mann-Whitney U test, all p < 0.001).

### EBV DNA quantity in nasopharyngeal brushings

**Figure 2:**
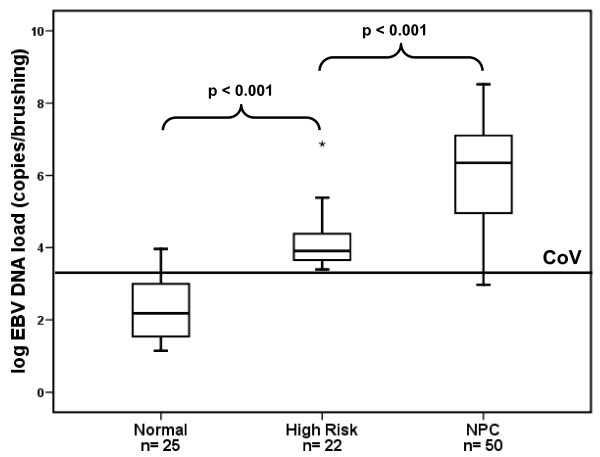
**Boxplot of (log) EBV DNA load in nasopharyngeal brushings by group (normal, high risk and NPC)**. The cut-off value was set at (log) 2,300 copies/brushing (3.362). There were highly significant variations between median in the three groups (one-way ANOVA, p < 0.001). Using Tukey analysis, the differrences between median in all groups are all significant. NPC = nasopharyngeal carcinoma, CoV = cut-off value.

Quantitative analysis of EBV DNA was determined in brushing samples from 50 NPC patients, 22 high risk individuals and 25 healthy subjects. The viral load was detectable in 49 NPC (98%) and all high risk isolates. In healthy subjects viral load was detectable in 4 cases (16%) and below the cut-off value (CoV) in 21 subjects (84%). There was a significant difference between the median log of EBV DNA quantity in NPC, high risk and normal population (p < 0.001) (Figure 2). The Post Hoc analysis using Tukey showed significant differences between NPC and high risk individuals as well as between NPC and normal EBV carriers (all p < 0.001).

### Nasopharyngeal carcinoma patient isolates showed frequent promoter methylation of 10 tumor suppressor genes

**Figure 3:**
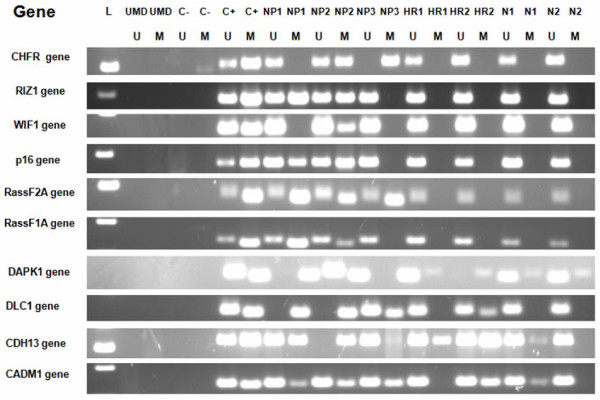
**Representative results of methylation specific PCR in the three groups**. NPC isolates showed more frequent methylated genes compared to high risk and normal isolates. L = marker, UMD = unmodied DNA, C- = negative control, C+ = positive control, NP = nasopharyngeal carcinoma, HR = high risk, N = normal, U = unmethylated alele, M = methylated alele.

For identification of methylation markers as complementary test for early detection of NPC, the frequency of promoter methylation of 10 TSGs was determined in NPC isolates. Identical methylation status in paraffin biopsy and the corresponding brushing samples was observed in 18 of 22 NPC cases, revealing an 81.8% overall agreement. Therefore methylation status in the NPC group was determined using either one of the two sample types. When determined in all isolates of NPC cases (n = 53) the frequency of promoter methylation of all individual genes ranged from 29.2-79.2% (table 1). Representative MSP pictures are shown in Figure 3. Fifty-two of 53 (98.1%) NPC cases exhibited aberrant promoter methylation in at least one of the genes studied. Three (5.6%) cases showed promoter methylation in all these genes. In one case (1.9%) none of the genes was found to be methylated.

**Table 1:** The frequency of gene promoter methylation of 10 tumor suppressor genes in NPC, high risk and normal subjects.

Gene	NPC group (paraffin and/or brushing)	High Risk Group (brushing)	EBV-normal carriers (brushing)
CHFR	31/53 (58.5%)	0/22 (0.0%)	0/25 (0.0%)
RIZ1	30/53 (56.6%)	0/22 (0.0%)	0/25 (0.0%)
WIF1	30/49 (61.2%)	4/22 (18.2%)	0/25 (0.0%)
p16	35/53 (66.0%)	6/22 (27.2%)	0/25 (0.0%)
RASSF2A	14/48 (29.2%)	0/22 (0.0%)	0/25 (0.0%)
RASSF1A	40/53 (75.5%)	0/22 (0.0%)	1/25 (4.0%)
DAPK1	42/53 (79.2%)	9/22 (40.9%)	12/25 (48.0%)
DLC1	40/52 (76.9%)	9/22 (40.9%)	16/25 (64.0%)
CDH13	41/53 (77.4%)	16/22 (72.7)	16/25 (64.0%)
CADM1	37/53 (69.8%)	8/22 (36.4%)	18/25 (72.0%)

In NPC group methylation status was determined either from paraffin or brushing DNA.

Frequency is presented in order of percentage in EBV-normal group from the smallest to the largest

For analysis of the possibility of TSGs contributing to early stage NPC the frequency of promoter methylation in high risk and normal individuals was determined (table 1). In the high risk population methylation was detected for some TSGs including CDH13 (72.7%), DAPK1 (40.9%), CADM1 (36.4%) and DLC1 (40.9%). The frequency of methylation of p16 and WIF1 gene was low (27.2% and 18.2% respectively), whereas methylation of CHFR, RIZ1, RASSF1A and RASSF2A gene was undetectable. In the normal population a high frequency of methylation was demonstrated for DAPK1 (48.0%), CDH13 (64.0%) and DLC1 (64.0%), whereas methylation of CADM1 (72%) was even higher than in NPC cases and high risk subjects. In healthy EBV carriers promoter methylation was very low for RASSF1A (4%) and undetectable for CHFR, RIZ1, WIF1, p16 and RASSF2A. The high methylation frequency in healthy individuals led us to evaluate methylation patterns in 11 peripheral blood mononuclear cells (PBMC) from Dutch healthy donors and 5 spontaneous lymphoblastoid cell lines (LCL) from Indonesian healthy donors. In these specimens the methylation frequency of the particular genes was detectable but was not as high as that of nasopharyngeal brushing from healthy donors (DAPK1 18.8%, DLC1 18.8%, CDH13 31.3% and CADM1 43.8%, respectively). In contrast, RASSF1A, RIZ1, CHFR, WIF1, p16 and RASSF2A showed no detectable methylation (data not shown).

### Confirmation of methylation status using Q-MSP

**Figure 4:**
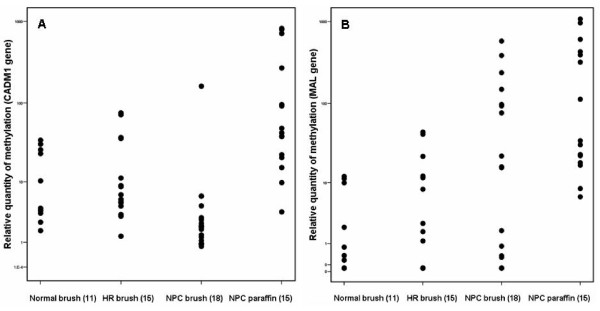
**Scatter diagram of relative quantity of methylation of (A) CADM1 and (B) MAL gene**. The Y axis is expressed in log scale. CADM1 (A) showed an insignificant variations between normal brushing and NPC brushing and between NPC brushing and NPC paraffin (one-way ANOVA using Tukey analysis p = 0.654 and 0.069 respectively). Thus Q-MSP confirmed the result of MSP. MAL (B) showed insignificant variations between normal brushing and NPC brushing (p = 0.070). Methylation level in NPC paraffin was comparable to NPC brushing (p = 0.069) but was significantly higher compared to normal brushing (p = 0.002) indicating that MAL methylation can be a promising marker for NPC detection. HR = high risk, NPC = nasopharyngeal carcinoma.

To determine whether the high frequency of CADM1 promoter methylation in the normal population was related to the level of methylation quantitative amplification was performed. Fifteen paraffin tissues and 18 brushings from NPC patients, 15 brushings from high risk and 11 brushings from normal individuals were selected for Q-MSP using Taqman. As shown in Figure 4A methylation levels of CADM1 were comparable between brushings of healthy controls, high risk subjects and NPC cases (p = 0.654 for healthy versus NPC and p = 0.641 for NPC versus high risk), confirming the MSP result. NPC paraffin yielded higher methylation levels compared to NPC brushing but the difference was not significant (p = 0.069).

### MAL promoter methylation showed good discrimination between NPC and normal subjects

In parallel to CADM1 we analysed MAL promoter methylation which was shown to be relevant in the viral oncogenesis of cervical cancer (Figure 4B). Better discriminating levels of methylation were demonstrated in NPC brushing compared to brushing from healthy EBV carriers (p = 0.070). The level of MAL methylation in NPC paraffin and NPC brushing was similar (p = 0.315) but in NPC paraffin it was significantly higher compared to brushing samples from healthy persons (p = 0.002). This indicates that MAL methylation status may be a promising additional marker for NPC detection.

### A panel of 5 methylated TSGs showed best discrimination between NPC and non-cancer individuals

In Indonesian NPC cases all 10 TSGs tested with MSP showed high frequency of promoter methylation. However when comparing the three study groups, not all TSGs allowed discrimination of NPC cases from non-NPC individuals. Thus, defined genes were selected as NPC markers with high methylation frequency including CHFR, RIZ1, WIF1, p16 and RASSF1A. NPC isolates showed methylation detection of 58.5-75.5% for these genes compared to almost undetectable frequency in normal EBV carriers and partly methylated in the high risk group (table 1). By using this panel of 5 markers the NPC detection rate was improved compared to the individual markers. The sensitivity and specificity of using at least one of the markers (analysed in 50 NPC cases versus 25 normal subjects) are 98% and 96%, respectively. By using 2 markers with the highest frequency (RASSF1A and p16 gene), the sensitivity and specificity of at least one of both are 91% and 96%, respectively.

### Correlation of methylation status with other parameters

**Figure 5:**
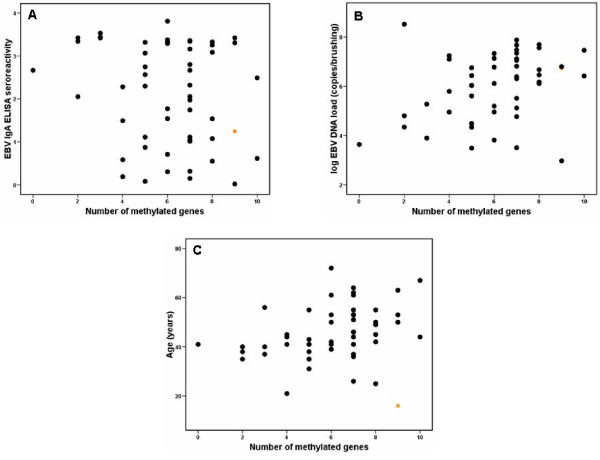
**Scatter diagram of (A) ELISA seroreactivity on 53 NPC sera, (B) log EBV DNA load on 50 NPC brushings and (C) age of 53 NPC cases versus number of methylated tumor suppressor genes**. The correlation with methylation rate is significant but weak for EBV DNA load (Pearson correlation r = 0.322, p = 0.023) and insignificant for ELISA seroreactivity (r = -1.88 p = 0.117) and age (r = 0.270, p = 0.05). The latter becomes significant when excluding a young patient from a multiplex family (symbolized with star) (p = 0.007).

The frequency of methylation was tested in relation to EBV parameters and age. There was a significant correlation between the rate of methylation with EBV DNA load (r = 0.322, p = 0.023) (Figure 5B), but not with EBV IgA ELISA seroreactivity (r = -1.88 p = 0.117) (Figure 5A) and age (r = 0.270, p = 0.05) (Figure 5C). When considering one outlier, methylation has a significant correlation with age (r = 0.369, p = 0.007). The outlier was a young patient from a rare multiplex NPC family.

## Discussion

Methylation of promoter DNA is considered an important epigenetic event in NPC carcinogenesis [7-9] and has been proven promising for diagnosis of multiple type of tumors [18,19]. Since the profile of TSG promoter methylation may vary according to tumor type [20], this study defined epigenetic variability in NPC. Aberrant methylation of five TSGs was identified as independent marker for early detection of NPC with added value to EBV IgA serology and DNA load. For a developing country like Indonesia such a test should be economical with simple but well standardized technology suited for screening in a large population. Therefore qualitative MSP is proposed, being less labor-intensive and inexpensive compared to other assays such as Q-MSP or bisulfite sequencing.

We selected a set of markers that have been evaluated in various NPC populations. Since methylation changes have been reported to occur early in carcinogenesis [7], epigenetic markers are potentially relevant as early indicators of subclinical presence of NPC. Our present study demonstrated that the promoter methylation frequency of individual TSGs is variable in NPC, confirming previous publications [10-16]. The methylation frequency of single genes varied from 29.2% (RASSF2A gene) to 79.2% (DAPK1 gene). This frequency is comparable to the one presented in other studies except for WIF1 [12] and RASSF2A [14] where the frequency in the Indonesian samples was much lower (61.2% and 29.2%, respectively).

For particular genes (DAPK1, CADM1, CDH13 and DLC1), the methylation frequency is not only high in NPC cases, but also in the high risk group and normal EBV carriers, thus limiting their diagnostic value. The high frequency of methylation has not been reported before in other healthy control populations, mostly showing a frequency below 10% [15,16,21,22]. However, the occurrence of methylation in normal tissue was confirmed in PBMCs and LCLs obtained from healthy individuals. In CADM1, the high frequency of methylation detected by MSP was explained by frequent but low level methylation in both normal and NPC brushing using Q-MSP. This suggested that CADM1 is not a good marker for the early detection of NPC. Similarly DAPK1, CDH13 and DLC1 could be considered as non-specific markers.

A panel of methylation markers consisting of CHFR, RIZ1, p16, WIF1 and RASSF1A is proposed as a complementary test for early NPC detection. These five TSGs showed high methylation in NPC cases and not more than 4% in the regional healthy EBV positive controls providing good differentiation between cancer cases and normal controls. Detection of aberrant methylation in at least one of this panel showed a rate of 98% in NPC group compared to individual markers. In particular, detection of aberrant methylation of p16 and RASSF1A may provide more simple testing with specificity and sensitivity above 90%.

Pilot testing of MAL methylation using Q-MSP revealed this gene as an additional discriminator between NPC and normal controls. As explored previously in cervical [18] the MAL gene may play a specific role in viral-epithelial malignancies. Since the MAL gene has not been analysed in NPC before, this report is the first to describe it as a promising marker for further investigation.

Comparing paraffin tissue and brushing samples of NPC cases revealed a good detection of methylation for both sample types, supporting the results of Sun *et al*. [16]. Nasopharyngeal brushing is proposed for sampling in population screening studies considering it is convenient and simple. When determined only on brushing samples, methylation of any marker of the selected genes was detected in 39 out of 41 NPC cases (95.1%). This is comparable to the assessment using either paraffin or brushing which detected methylation in 98% of NPC. Moreover, this minimal invasive procedure yielded high PPV (>90%) compared to biopsy and reproducible high yields of cellular DNA (10^6^-10^7 ^genome copies/brush). Nasopharyngeal brushing is also an excellent sampling method for monitoring EBV DNA load and EBV mRNA expression, each having good diagnostic value [23].

Our present study did not demonstrate a significant difference between methylation frequency in early and late stage NPC cases (data not shown). To evaluate the capacity of epigenetic markers to detect preclinical disease, a longitudinal study is currently in progress in the local high risk population. Compared to the baseline observation, this cohort revealed an increased frequency of methylation of p16 and RIZ1. The presence of p16 methylation supports previous reports showing p16 gene silencing in precancerous lesions of NPC. However our data do not confirm RASSF1A methylation in premalignant tissues [7]. This indicates that epigenetic changes leading to malignancy in the Indonesian population may differ from other populations. Which gene silencing event initiates NPC development and which contributes to further progression of the multistep carcinogenic process remains to be investigated.

The correlation found between methylation status and viral DNA load in Indonesian NPC cases indicates a link between epigenetic events and EBV infection. The appropriate level of DNA load in our NPC samples may directly reflect a (pre)malignant process. This is in agreement with a previous data on matched NPC tumor and adjacent tissues. The nearer to the NPC tissue, the higher the levels of gene promoter methylation and EBV DNA load found [11]. More recent studies implied the role of EBV genes in epigenetic silencing either through activation of DNA methyltransferases [24] or interaction with transcriptional repression [25].

Factors known to contribute to methylation alterations include age, diet and lifestyle [26,27]. Regarding the factor of age, our result on Indonesian NPC showed a tendency for methylation frequency to increase with age. This reflects accumulative epigenetic events under the influence of environmental exposures that may increase with time. Considering the factor of diet, our observation in the local population demonstrates that diet might also predispose one towards cancer development. Formaldehyde, boric acid, Rhodamine B, and yellow Metanyl are among many reported chemicals found in the food of local markets in Indonesia [28]. Persistent exposure to environmental toxins is indicated as an initial step of epigenetic alteration preceding cancer development [29-31]. This might explain the high frequency of methylation of certain genes observed in the Indonesian healthy population. The potential relationship between carcinogenic exposure and promoter methylation status is subject to further study. Analysis of the methylation status of selected genes is proposed as additive test for NPC risk assessment using EBV-based assays in primary screening. The epigenetic markers described here provide complementary information in a subgroup of high risk individuals with aberrant EBV IgA seroreactivity and elevated viral load. In a case-finding approach, screening subjects presenting with symptoms suspicious of NPC, this approach may identify those subjects at highest risk of NPC development.

## Conclusion

Indonesian NPC cases showed more frequent methylated TSGs compared to high risk and normal individuals implicating epigenetic changes in NPC development and suggesting their utility for NPC identification. DNA promoter methylation of defined sets of genes may serve as a complementary test for early NPC detection in combination with EBV-based markers. Such a test should include p16 and RASSF1A possibly combined with CHFR, RIZ1, WIF1 and MAL.

## Materials and methods

### Patients and biological samples

Paraffin-embedded tissue from 34 NPC patients before treatment were selected from the pathology archives of Dr. Sardjito Teaching Hospital Yogyakarta [23]. Corresponding nasopharyngeal brushings were collected from 22 of these patients. Single nasopharyngeal brushing samples from 19 NPC cases were obtained yielding 53 overal NPC cases. NPC cases were histologically confirmed as undifferentiated NPC (WHO type 3). Presence of EBV was confirmed by EBV-encoded small RNA (EBER) detection in all cases [23]. Staging was done according to the AJCC 2004 criteria with 44 patients (80%) at late stage NPC (3 or 4) and 9 patients at early stage (1 or 2). Twenty-two individuals with chronic symptoms suggestive for early NPC [4] and having elevated EBV markers were selected as the high risk population. Twenty-five asymptomatic EBV carriers were recruited consisting of 9 Dutch and 16 Indonesian subjects. All participants were recruited with informed consent to give blood and nasopharyngeal brushings.

### DNA extraction and bisulfite modification

Genomic DNA from nasopharyngeal brushings was isolated using silica-based extraction [32] (Basic kit, BioMerieux, Boxtel, The Netherlands). DNA isolation from paraffin-embedded tissue was performed using the Chelex method [33]. Of each DNA sample 500 ng was used for bisulfite treatment using a commercially available DNA modification kit (EZ DNA Methylation Kit™; Zymo Research, Orange, CA, USA).

### EBV markers

EBV specific IgA reactivity was assessed in all sera by a synthetic peptide based enzyme-linked immunosorbent assay (ELISA) for immunodominant epitopes derived from Epstein-Barr virus nuclear antigen 1 (EBNA-1) and viral capsid antigen (VCA)-p18 (further referred to as EBV IgA ELISA). The ELISA CoV was 0.354 as previously defined [34]. EBV DNA load in nasopharyngeal (NP) brushings was determined by quantitative LightCycler (LC) real-time PCR targeting a 213 bp conserved region of EBNA-1 [35]. The CoV was set on 2,300 copies/brushing presenting the mean+3*SD of a cohort of controls [23].

### Methylation-specific PCR (MSP) and quantitative MSP (Q-MSP)

MSP targeted regions within promoters of 10 TSGs including CHFR [36], RIZ1 [37], WIF1 [12], p16 [38], RASSF1A [39], RASSF2A [40], DAPK1 [41], DLC1 [15], CDH13 [42] and CADM1 [43]. The sequences of PCR primers specific for unmethylated and methylated alleles of all targets were identical as previously published. FastStart Taq PCR and Amplitaq Gold (Roche Diagnostics Netherlands BV) were used for ampification. The EBV positive Burkitt's lymphoma cell line (Namalwa), EBV negative nasopharyngeal carcinoma cell line (HONE-1), cervical cancer cell lines (CaSki, SiHa), human lung adenocarcinoma cell line (Calu-6), human colon carcinoma cell line (RKO) and primary human foreskin keratinocytes (EK) were included as positive and negative PCR controls for either unmethylated or methylated amplification. For these cell lines methylation status of individual TSGs was known [10-12,15,16,44]. As negative controls, H_2_O, bisulfite modified DNA of primary keratinocytes and unmodified methylated DNA from cell lines or brushing samples were included.

Quantitative-MSP (Q-MSP) was conducted using primer sets targeting the CADM1 and the MAL gene promoter. Amplicons were detected and quantified using Taqman probes. Primer/probe sequence for CADM1 is (forward) ATTTTATTACTTCTTCGTTCGGGT, (probe) ACCTACCTCAAACTAACGACGTTAACTACCTCCGA and (reverse) CTCGACAACACTACA CTCGCC [45]. The Q-MSP amplicon largely overlaps the conventional MSP amplicon (CADM1 region M9) [43]. Primer/probe sequences for the MAL promoter were described previously [18]. Q-MSP was set up using QuantiTect Probe PCR Kit master mix (Qiagen, Westburg, Leusden, The Netherlands). For each Q-MSP a standard curve consisting of serial dilutions of bisulfite-treated DNA of the SiHa cervical cell line was used. The housekeeping gene β-actin was applied as an internal reference. To determine the relative quantity of methylation, ratios between methylated CADM1 or MAL DNA versus β-actin DNA was performed (average quantity of methylated CADM1/MAL DNA/average DNA quantity for β-actin × 1000).

### Statistical analysis

Differences of methylation status between the three groups versus other diagnostic parameters were analysed using one-way analysis of variance (ANOVA) (EBV DNA load) or Mann-Whitney U test (ELISA seroreactivity). Differences of methylation status in Q-MSP were analysed using one-way ANOVA. The correlation between methylation status and ELISA seroreactivity, EBV DNA load and age was assessed using the Pearson correlation test. p < 0.05 was considered significant. All statistical calculations were performed using SPSS version 15.0.

## Competing interests

The authors declare that they have no competing interests.

## Authors' contributions

JMM conceived the study. SHH carried out the molecular work, statistical analysis, data interpretation and wrote the initial draft of the manuscript. SRI and LPLI participated in clinical sampling. H participated in sample selection and pathological expertise. SD carried out quantitative methylation-specific PCR and participated in data interpretation. SMH, RDS and AEG supervised the molecular work, participated in data interpretation and edited the manuscript. JMM and SHH had the primary responsibility for interpreting the data and editing the final manuscript. All authors provided comments of various drafts, participated in direction setting discussions and reviews and have read and approved the final version.
